# Inhibition of c-Abl Kinase Activity Renders Cancer Cells Highly Sensitive to Mitoxantrone

**DOI:** 10.1371/journal.pone.0105526

**Published:** 2014-08-22

**Authors:** Kemal Alpay, Mehdi Farshchian, Johanna Tuomela, Jouko Sandholm, Kaappo Aittokallio, Elina Siljamäki, Marko Kallio, Veli-Matti Kähäri, Sakari Hietanen

**Affiliations:** 1 Department of Obstetrics and Gynecology and Joint Clinical Biochemistry Laboratory of Turku University Hospital, Medicity Research Laboratory, University of Turku, Turku, Finland; 2 Department of Dermatology and MediCity Research Laboratory, University of Turku and Turku University Hospital, Turku, Finland; 3 Department of Cell Biology and Anatomy, University of Turku, Turku, Finland; 4 Cell Imaging Core, Turku Centre for Biotechnology, University of Turku and Åbo Akademi University, Turku, Finland; 5 VTT Health, VTT Technical Research Centre of Finland, Turku, Finland; Louisiana State University Health Sciences Center, United States of America

## Abstract

Although c-Abl has increasingly emerged as a key player in the DNA damage response, its role in this context is far from clear. We studied the effect of inhibition of c-Abl kinase activity by imatinib with chemotherapy drugs and found a striking difference in cell survival after combined mitoxantrone (MX) and imatinib treatment compared to a panel of other chemotherapy drugs. The combinatory treatment induced apoptosis in HeLa cells and other cancer cell lines but not in primary fibroblasts. The difference in MX and doxorubicin was related to significant augmentation of DNA damage. Transcriptionally active p53 accumulated in cells in which human papillomavirus E6 normally degrades p53. The combination treatment resulted in caspase activation and apoptosis, but this effect did not depend on either p53 or p73 activity. Despite increased p53 activity, the cells arrested in G2 phase became defective in this checkpoint, allowing cell cycle progression. The effect after MX treatment depended partially on c-Abl: Short interfering RNA knockdown of c-Abl rendered HeLa cells less sensitive to MX. The effect of imatinib was decreased by c-Abl siRNA suggesting a role for catalytically inactive c-Abl in the death cascade. These findings indicate that MX has a unique cytotoxic effect when the kinase activity of c-Abl is inhibited. The treatment results in increased DNA damage and c-Abl–dependent apoptosis, which may offer new possibilities for potentiation of cancer chemotherapy.

## Introduction

Chemotherapy in tumor treatment works mainly through causing DNA damage that induces a complex network of cellular responses ultimately leading to cancer cell death. At the core of the response are pathways that recognize the damage, halt the cell cycle, and enact the death cascade. In cancer therapy, radiotherapy and most chemotherapy agents function by directly damaging DNA or interfering with DNA replication. The DNA damage response of malignant and normal cells determines the efficacy and side effects of the treatment. The fate of the cell lies in the complex DNA repair pathways evoked by numerous types of DNA damage that can arise after genotoxic treatment [1]. Successful repair is critical for normal tissue to overcome the side effects of the therapy but in the tumor can result in treatment resistance. Cancer cells usually have accumulated mutations in genes involved in DNA repair, offering a variety of therapeutic opportunities for agents that modulate the remaining functional repair pathways. After DNA damaging treatment, damaged bases, mismatches, or DNA adducts are usually tolerated up to a certain quantitative threshold but can give rise to mutations if they remain unrepaired [2].

c-Abl inhibition has been recently proposed to lead to an altered DNA damage response [3]. c-Abl is a non–receptor tyrosine kinase that plays a role in differentiation, adhesion, cell division, death, and stress responses and binds to several proteins involved in apoptosis pathways [4]. The changes in c-Abl protein conformation vary, and the binding partners consequently differ [4]–[6]. Several proteins such as ATM, DNA-PK, BRCA1, and the transcription factors p73 and RFX1 interact with c-Abl [5]. Most notably, c-Abl has been reported to interact with the homologous recombination-repair protein Rad51, elevate [7] its expression at the gene level, and activate it by phosphorylation. Active c-Abl can be inhibited by the small molecule drug imatinib (Gleevec; STI-571), which was developed against the aberrant BCR/Abl fusion protein found in chronic myeloid leukemia (CML) [8]. In CML cells, the first exon of c-Abl is replaced by the BCR gene sequence, resulting in constitutively active c-Abl expression. This aberrant kinase activity results in enhanced proliferation, which can be inhibited with imatinib. Imatinib is an ATP-competitive inhibitor stabilizing inactive c-Abl conformation [8]. The kinase activity of c-Abl is increased after DNA damage and then increases the activity of Atm and Atr [9]. Treatment with imatinib decreases the level of elevated RAD51 involved in double-strand break (DSB) repair and sensitizes several cell types to chemotherapy [10]–[13]. Direct interaction has also been demonstrated between c-Abl and DNA-PK, which regulates non-homologous end joining [14].

The development of uterine cervical cancer is a multistep process that involves cervical mucosal cell transformation by oncogenic human papillomavirus (HPV) E6 and E7 proteins. E7 inactivates the cell cycle regulator pRb, inhibiting cell cycle arrest, while E6 inactivates the tumor suppressor protein p53, the key regulator of apoptosis and genotoxic stress response [15]. Because cervical cancer cells almost always carry wild-type p53, which is degraded by high-risk HPV, p53 was formerly regarded as completely non-functional in cervical cancer cells. However, the work of several groups has recently made evident that p53 inactivation may be reverted in HPV E6–carrying cells and that p53 status in cervical cancer cells is not equal to that of cancer cells with a mutated p53 gene [16]. We previously observed that chemoradiation reactivates p53 in cervical cancer cells and promotes cell death synergistically. However, when analyzed in detail, the p53 protein may either enhance or inhibit the cytotoxicity of the chemotherapy drug [17], [18]. Mouse embryonic fibroblasts null for c-Abl are defective in p53 phosphorylation and resistant to death after genotoxic damage. Inhibition of c-Abl by imatinib diminishes hydroxyurea-induced p53 phosphorylation [9]. We hypothesized that the active p53 may enhance DNA repair and thus wanted to study the effect on cell death of repair modulation. c-Abl is generally believed to relay pro-apoptotic signaling from Atm and Atr to p53 and p73, among other targets [3]. We studied here the effect of c-Abl inhibition on p53 activity in HPV-positive cells and how it relates to the damage and death responses.

A comprehensive panel of drugs representing alkylating agents, platinum drugs, and topoisomerase I and II inhibitors was studied together with imatinib in cervical cancer cells carrying HPV and in HPV-negative cell lines. We report here that c-Abl inhibition by imatinib in combination with MX genotoxic treatment results in impaired DNA repair, abrogation of the G2 phase checkpoint, and massive apoptosis.

## Materials and Methods

### Cell lines and cytotoxicity assays

The human cervical cancer cell lines SiHa (HPV 16+), CaSki (HPV16+), and HeLa (HPV 18+), breast cancer cell line MCF7, and vulvar cancer cell line A431 were obtained from the American Type Culture Collection (Manassas, VA, USA). The primary human fibroblasts have been described before [19]. The cells were grown as monolayers in DMEM supplemented with 10% fetal bovine serum, 2 mM L-glutamine, non-essential amino acids (Euroclone, Wetherby, UK), and 50 µg/ml gentamycin (Calbiochem, San Diego, CA, USA). The HeLa p53 reporter cell line, carrying the p53 reporter plasmid ptkGC3p53-luc, has been described previously [18]. Despite the presence of HPV E6, even the HPV-positive cell lines show some p53 activity after genotoxic stress, but the activity can be degraded by dominant-negative p53 (DDp53) or ectopic E6 driven by a strong promoter. The SiHa DDp53, SiHa CMV, HeLa DDp53, HeLa CMV (empty vector), and SiHa E6 cell lines have also been described previously [18].

The dominant-negative p53–expressing HeLa cell line (HeLa DD) was derived by transfecting the parental cell line with a plasmid that expresses a truncated mouse p53 containing amino acid residues 1–14 and 302–390 under the control of the CMV promoter. Stable transfectants were selected with 0.8 mg/ml G418.

In short-term cell viability assays, 1–2×10^4^ cells per well (depending on cell line) were seeded into 96-well plates, and the medium was replaced with drugs diluted with medium. The cell viability was measured by WST-1 agent (Roche, Mannheim, Germany) or MTT agent (Sigma-Aldrich Inc., St. Louis, MO, USA), and absorbance was measured at 450 nm (Multiskan plate microreader; Labsystems, Finland) or at 570 nm (Tecan multi-plate reader; Tecan, Switzerland), respectively.

For clonogenic growth assays, SiHa and HeLa cells were seeded into 6-well plates 48 hours before treatment. The cells were exposed to treatment for 6 hours and then trypsinized and suspended in fresh medium and seeded into 6-well plates with 3 µM imatinib. SiHa cells were incubated for 14 days and HeLa cells for 7 days. Following incubation, cells were fixed with 1∶1 acetone–methanol and stained with Giemsa (Merck, Whitehouse Station, NJ, USA). Then clones were either counted manually or analyzed as described previously [20]. Caspase 3/7 activity of cells undergoing apoptosis was determined using the Apo-ONE Caspase-3/7 homogenous caspase assay (Promega).

### Reagents, drugs, and antibodies

The chemotherapy compounds mitoxantrone (MX) (Wyeth-Lederle, Finland), doxorubicin (DXR) (Nycomed, Roskilde, Denmark), cyclophosphamide (Orion Pharma, Espoo, Finland), topotecan (GlaxoSmithKline, Uxbridge, Middlesex, UK), etoposide (Pfizer), cisplatin (Bristol-Myers Squibb, Princeton, NJ, USA), docetaxel (Aventis), and carboplatin (Bristol-Myers Squibb, Princeton, NJ, USA) were stored and prepared as described [17]. Imatinib was a gift from Novartis Pharmaceuticals (Basel, Switzerland). The stock solution of imatinib at 200 mM was prepared by dissolving the compound in DMSO. The c-kit and PDGF-α and β receptor blocker AG1296 was purchased from Calbiochem (cat. no. 658551). PDGF-BB was purchased from Sigma-Aldrich. The following antibodies were used for Western blotting: mouse monoclonal DO-1 for p53 (Santa Cruz Biotechnology, Santa Cruz, CA, USA), rabbit polyclonal anti-GADD45α (Cell Signaling, cat. no. 3518), rabbit polyclonal anti-phospho-PDGF β receptor (Cell Signaling, cat. no. 3161), monoclonal mouse anti-RAD51 (Invitrogen, Carlsbad, CA, USA; cat. No. 35–6500), monoclonal mouse anti-cyclin B1 (BD Biosciences, cat. no. 554178) and monoclonal anti-p73 (Santa Cruz Biotechnology, Santa Cruz, CA, USA). The RNA was isolated using the RNeasy kit (Qiagen, Hilden, Germany).

### Short interfering RNAs and transfections

The c-Abl short interfering RNAs (siRNAs) were obtained from Invitrogen (Stealth, Carlsbad, CA, USA; cat. no. 1299003), and non-targeting siRNA (Qiagen, cat. no. 1027281) was used as control. Transfection of the cells was performed with 75 nM of three individual siRNAs targeting c-Abl and control siRNA using the siLentFect Lipid Reagent (Bio-Rad).

### p53 reporter assay

Stable ptkGC3p53luc-bsd SiHa, CaSki, and HeLa cell lines as well as the composition of the p53 reporter plasmid ptkGC3p53-luc have been described earlier [17]. The cells were seeded into 96-well plates (10^4^ cells/well). After allowing for cell attachment for 24 hours, the treatments were begun for the indicated durations. The living cells in each well were determined colorimetrically with the WST-1 assay. Thereafter, the cells were rinsed with PBS and overlaid with 100 µl of a mixture containing 50% PBS and 50% Bright-Glo luciferase assay reagent (Promega, Madison, WI, USA). The luciferase activity was quantified with the aid of a hybrid capture luminometer (Digene, Gaithersburg, MD, USA). Luciferase readings were divided by WST-1 value to obtain normalized reporter activity.

### Western blotting

The cells were harvested using 200 µl of standard 1×SDS sample buffer. The resulting whole cell extracts were boiled and then separated by 10% SDS-PAGE and transferred to Immobilon-P polyvinylidene fluoride membranes (Millipore, Billerica, MA, USA). The membranes were probed with the indicated primary antibodies, and secondary detection was done with anti-mouse horseradish peroxidase (HRP) (GE Healthcare, NJ, USA), anti-rabbit HRP (DAKO, Glostrup, DK), and ECL (GE healthcare, NJ, USA). Beta-actin was used as a loading control. The Western blot films were digitized with a ChemiDoc MP gel analysis platform (Bio-Rad, Hercules, CA, USA). and analyzed with Fiji (ImageJ) ver 1.47q (Wayne Rasband, NIH, USA) using the Gels option.

### Flow cytometry

Cell cycle analysis was performed by flow cytometry. Cells were harvested with trypsinization together with floating non-viable cells. The cells were washed once with PBS and suspended in sodium citrate buffer (40 mM Na-Citrate, 0.3% Triton X-100, 0.05 mg/ml propidium iodide, PBS) 20 minutes prior to analysis. Cell cycle analysis was performed using FACSCalibur (Becton Dickinson, CA, USA) and CellQuest Pro software (Becton Dickinson). Cell cycle and apoptosis analyses were performed with ModFit LT (Verity Software House, Inc., Topsham, ME, USA) and Flowing Software ver. 2.5 (Mr. Perttu Terho, Turku Centre for Biotechnology, Finland, www.flowingsoftware.com), respectively. To further analyze the apoptosis induction after MX and MX + imatinib treatment HeLa cells were grown on 6-well plates and treated with indicated drugs for 24 and 48 hours. Medium and cells were collected and the samples were stained with Annexin-V-FITC kit (ab14085; Abcam, Cambridge, UK) according to manufacturer's instructions. Data were acquired with a FACSCalibur flow cytometer, and analyzed with Flowing Software.

### Real-time quantitative reverse transcription PCR

The RT-PCR method has been described before [18]. The primers were HPV 18 E6, forward, 5′-TGGCGCGCTTTGAGGA-3′, and reverse, 5′-TGTTCAGTTCCGTGCACAGATC-3′; and EF1α, forward, 5′-CTGAACCATCCAGGCCAAAT-3′, and reverse, 5′-GCCGTGTGGCAATCCAAT-3′. The amounts of HPV 18 E6 transcripts were normalized against the readings for EF1α.

### Comet assay

DNA damage was studied with a single cell gel electrophoresis kit (Trivigen, Gaithersburg, MD, USA). The assay was performed in alkaline conditions to detect both single- and double-stranded DNA damage. The single-cell gel electrophoresis of DNA was performed as described by the manufacturer. Images were captured using an Olympus BX60 fluorescence microscope (Zeiss AxioVert 200 M) at ×20 magnification.

### Time-lapse microscopy

Images were captured in one hour interval for 72 hours with IncuCyte ZOOM kinetic imaging system (Essen Bioscience, Michigan, USA). Representative wells were selected and movies were constructed with ImageJ ver 1.47d (Wayne Rasband, NIH, USA). Bar represents 200 µm.

### Statistics

To evaluate differences between groups, we used Student's t-tests. A p value below 0.05 was considered to indicate statistical significance.

## Results

### Inhibition of c-Abl by imatinib potentiates the cytotoxic effect of mitoxantrone in cancer cells but not in primary fibroblasts

**Figure 1:**
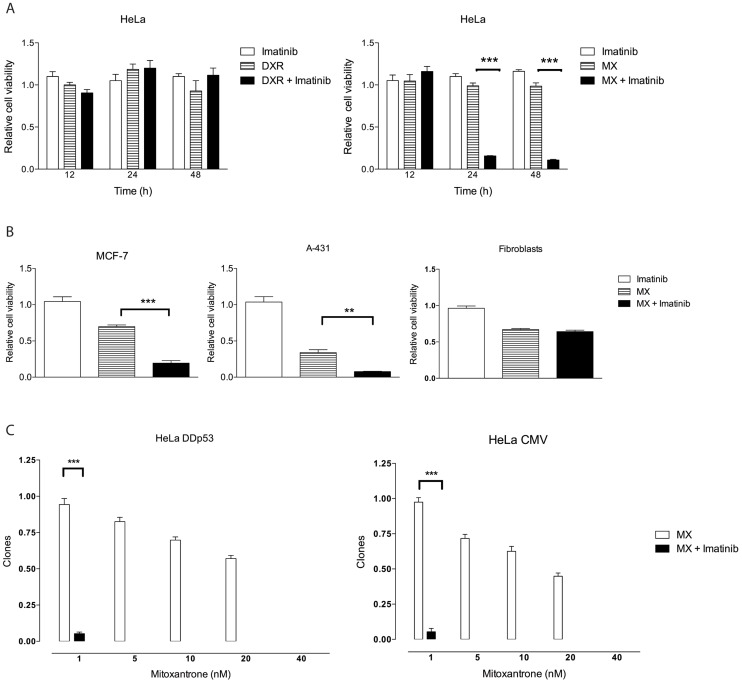
Imatinib increases cytotoxicity of mitoxantrone (MX) but not doxorubicin (DXR) in HeLa cells and this effect is not specific for cervical cancer cell lines or dependent on p53 status. (A) Human cervical cancer (HeLa) cells were treated with MX (0.6 µM), DXR (0.8 µM), imatinib (10 µM) or their combinations. WST cell viability assay was performed at 12 h, 24 h and 48 h. The results were normalized with cell number in medium only containing wells. *** p<0.001 independent samples T-test. (B) Both A-431 vulvar carcinoma cell line which has a missense mutation in the p53 gene and MCF-7 breast cancer cell line which has a wild type p53 gene show an enhanced effect when MX and imatinib are combined. Imatinib does not increase the cytotoxicity of MX in primary fibroblasts. Measurements were performed at 48 h using WST cell viability assay. ** p<0.01, *** p<0.001 independent samples T-test. (C) Clonogenic assay. Imatinib enhances MX cytotoxicity both in in HeLa CMV cell line with wild-type p53 and empty vector and HeLa DDp53 cell line carrying a dominant negative p53. Cells were treated with each drug for 12 h. Then, medium was replaced with fresh medium without drugs. The concentration of imatinib was 3 µM whereas MX was used in different concentrations ranging from 1 nM to 40 nM. Clones were counted under microscope. Experiment was done in triplicate, mean ± SD. *** p<0.001.

The potency of imatinib in enhancing cytotoxicity was screened in a large panel of chemotherapy drugs. In the short-term cytotoxicity assays, imatinib alone was not cytotoxic to any of the cell lines at 3 µM to 10 µM. When HeLa, CaSki, and SiHa cells were treated with the topoisomerase I inhibitors (topotecan and etoposide), nucleoside analogues (gemcitabine, fluorouracil, and cytarabine), alkylating agents (cyclophosphamide and dacarbazine), or cisplatin, imatinib did not enhance cytotoxicity (data not shown). Neither did it affect anthracycline-(doxorubicin,DXR) treated cells (Figure 1A). The effect of carboplatin was enhanced two fold by addition of imatinib for 48 hours (data not shown). In contrast to all the other chemotherapy drugs tested, imatinib showed a dramatic effect together with mitoxantrone (MX), a topoisomerase II inhibitor (Figure 1A). The effect of imatinib was equal between 3 µM and 10 µM indicating that the kinase activity is blocked in the studied cell lines even at 3 µM.

The enhanced effect was also seen in CaSki and SiHa cells although to a lesser extent (Figure S1). The cell lines that we primarily wanted to test were derived from HPV-positive cervical cancer. However, the effect appears to not have been restricted to these cells. The drugs were tested with two cancer cell lines of different origin: The vulvar carcinoma cell line A431 has a missense mutation in the p53 gene, and the MCF7 breast cancer cell line has a wild-type p53. The survival was strikingly similar to the HPV-positive cell lines (Figure 1B). In contrast, primary non-transformed fibroblasts were not more sensitive when imatinib was added to MX treatment (Figure 1B). This result indicates that the observed effect is not restricted to HPV-positive cancer cells but can occur more broadly regardless of p53 status. However, non-transformed cells may exhibit resistance to this treatment.

The enhanced effect of imatinib was also seen in native and differently modified HeLa and SiHa cells in a manner independent of either p53 or E6 (Figure S2). MX was further studied in clonogenic growth assays to monitor the long-term recovery capacity of the cells. MX concentrations as low as 1 nM together with 3 µM imatinib inhibited HeLa cell clonal growth completely, both p53-null and empty vector–carrying cells (Figure 1C). In SiHa cell lines, 3 µM imatinib alone did not affect clonal growth. The sensitivity of CaSki cells for imatinib addition was between that of SiHa and HeLa cells (Figure S3).

### Mitoxantrone induces caspase 3/7, which is enhanced by blocking c-Abl with imatinib

**Figure 2:**
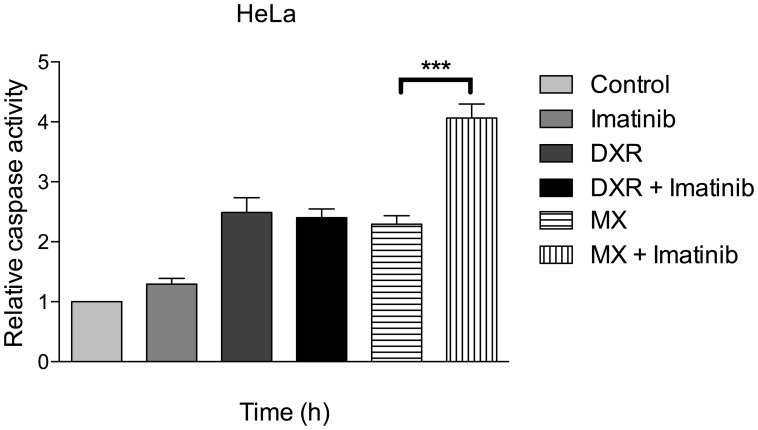
Combination of MX and imatinib activates caspase cascades. HeLa cells were treated with indicated drugs for 48 h. Then, fresh medium was replaced and caspase 3/7 activity was measured using ELISA assay. Experiment was done in triplicate, mean ± SD. *** p<0.001 T-test.

The treatment with MX increases the release of caspase 3 and 7 from the mitochondria indicative of apoptosis. Imatinib alone is not able to alter these levels (Figure 2). Cells treated with 0.6 µM MX and 0.8 µM DXR increased the caspase activity 2.5 fold, whereas the MX + imatinib combination treatment increased the activity 4 fold. Imatinib did not increase the activity induced by DXR alone.

### Inhibition of c-Abl kinase activity increases DNA damage in MX-treated cells

**Figure 3:**
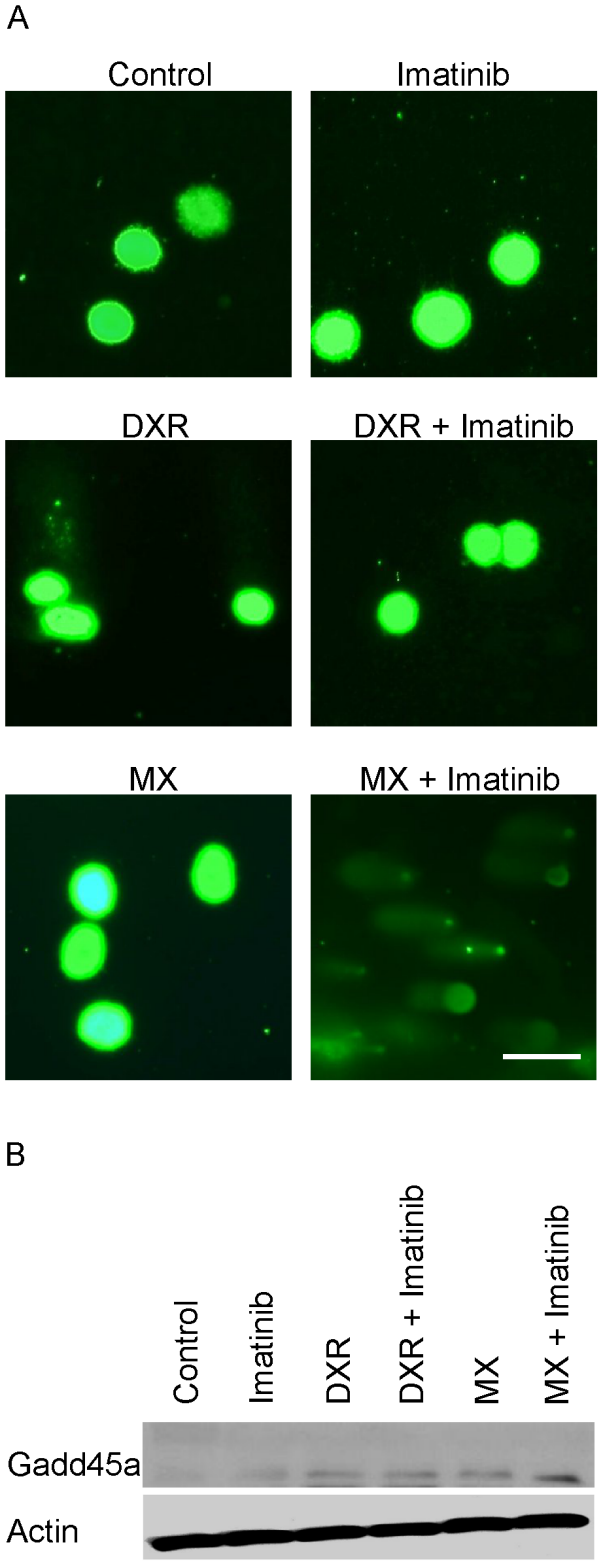
Imatinib increases DNA-damage induced by MX but not by DXR. (A) DNA damage in the HeLa cells was studied using Comet assay after treatment with MX (0.6 µM), DXR (0.8 µM) and imatinib (5 µM), or their combinations. Cometting (tailing) indicates DNA damage. (B) Imatinib combined with MX increases the level of GADD45α. Western blot of GADD45α protein levels from whole cell lysates. HeLa cells were treated with MX (0.6 µM), DXR (0.8 µM), imatinib (5 µM), or their combinations for 30 h.

**Figure 4:**
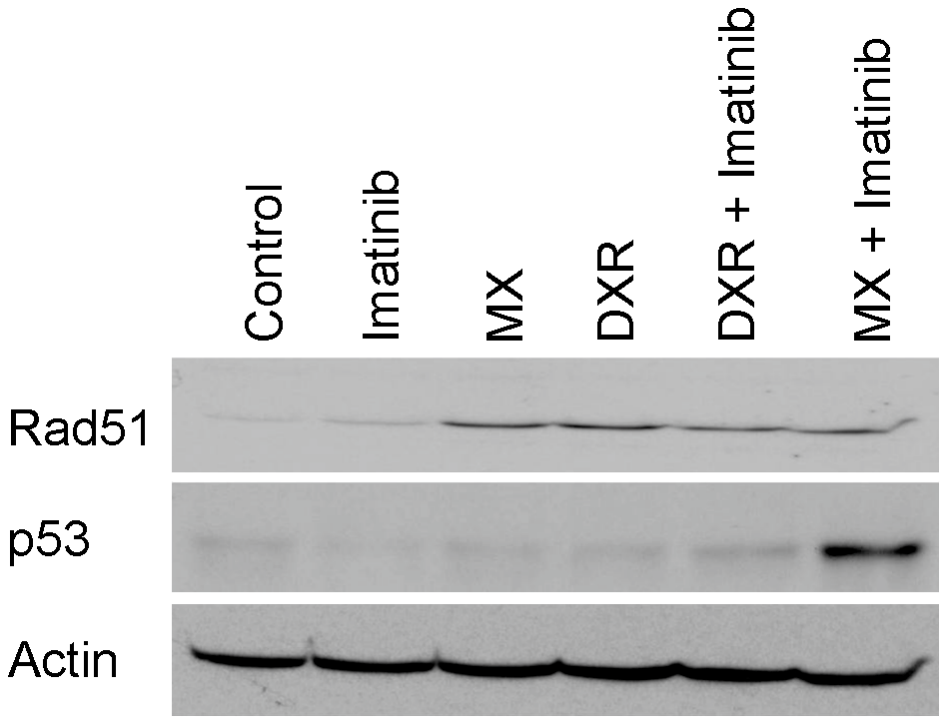
Imatinib increases p53 levels when combined with MX, but not with DXR. Western blot of p53 and RAD 51 in HeLa cells. Imatinib combined with MX increases p53 protein levels in HeLa cells. RAD51 level was slightly reduced in cells treated with imatinib and MX. Cells were treated with MX (0.6 µM), DXR (0.8 µM), imatinib (5 µM) or their combinations for 30 hours.

Adding imatinib to MX increased comet tailing in HeLa cells whereas imatinib alone did not have any effect (Figure 3A). Imatinib did not induce tailing in DXR-treated cells. The amount of GADD45α protein increased slightly even in the whole cell lysates with MX + imatinib (5 fold, Figure 3B). This increase seems to be regulated at the transcriptional level because we also have observed a marked increase in GADD45α transcript levels after the combination therapy in microarray RNA analyses (unpublished data). Like p53 protein accumulation in cell stress, GADD45α transcription increases under stress, including with DNA damage. Both DXR and MX increased RAD51 levels (10 fold), and imatinib pretreatment inhibited the increase equally (20%) but enhanced the accumulation of p53 when added to MX (13 fold, Figure 4).

### Mitoxantrone-induced p53 activation is enhanced by inhibiting c-Abl kinase activity

The chemotherapy drugs used in this study induce various forms of DNA damage, which p53 in the target cells senses. p53 is activated in cervical cells despite the degradation activity of E6 [16], [19]. We measured p53 reporter activity in HeLa, CaSki, and SiHa cell lines with DXR, MX, and cisplatin. When the drugs were compared, cisplatin induced the reporter more than MX in HeLa and CaSki cells but similarly in SiHa cells, and 0.6 µM MX alone did not activate the reporter at all in HeLa cells at 48 hours. DXR induced the reporter more than cisplatin and MX in all cell lines (Table 1). Imatinib alone did not significantly alter the activity in any of the cell lines. Adding imatinib to cisplatin slightly reduced the activity, but there was no effect for DXR. In contrast, adding imatinib to 0.6 µM MX increased the activity in all cell lines, by 50% in SiHa cells but four fold in HeLa cells and eight fold in CaSki cells. The limitation of this approach is that a direct comparison between cell lines cannot be made because of different amounts of integrated reporter plasmid. Consistent with the reporter assays, p53 protein level was also significantly increased in Western blot analyses (Figure 4). We saw no decrease in p53 level with imatinib alone, results that were in accordance with the reporter analysis outcomes.

**Table 1:** p53 reporter activity changes in HeLa, SiHa and CaSki cells.

	Hela	SiHa	CaSki
Cisplatin	2.7±0.2	3.6±0.3	14.7±0.6
Cisplatin + Imatinib	1.9±0.1	2.6±0.1	10.3±0.0
Doxorubicin	4.3±0.2	9.5±0.3	32.6±0.6
Doxorubicin + Imatinib	4.2±0.23	8.6±0.35	31.1±0.4
Mitoxantrone	0.9±0.1	3.8±0.1	3.0±0.2
Mitoxantrone + Imatinib	4.1±0.0	4.7±0.2	24.0±0.2
Imatinib	1.0±0.0	1.2±0.1	1.2±0.1

The results are given in fold-change. The concentrations used were MX 0.6 µM, 1 µM and 1 µM in HeLa, SiHa and CaSKi cell lines, respectively; DXR 0.8 µM, 1.4 µM and 1 µM in HeLa, SiHa and CaSKi cell lines, respectively; Cisplatin 40 µM, 70 µM and 40 µM in HeLa, SiHa and CaSKi cell lines respectively. Imatinib 10 µM in each cell line.

p73 is a member of the p53 protein family and interacts with c-Abl directly and can also induce apoptosis. The p73 protein levels did not change after the treatments (Figure S4).

### Imatinib does not alter HPV E6 levels

Most of the chemotherapy drugs reduce the amount of E6 mRNA [17]. We wanted to know whether imatinib causes reduction in E6 expression in HeLa cells either alone or combined with MX. We found that 1 µM MX decreases E6 mRNA levels in HeLa cells by approximately 50% and that 5 µM imatinib alone does not reduce the level of E6 mRNA in these cells. A combination of 5 µM imatinib and 1 µM MX did not reduce the level of E6 mRNA in HeLa cells more than 1 µM MX alone.

### Imatinib abrogates S-phase arrest caused by MX and increases apoptosis

**Figure 5:**
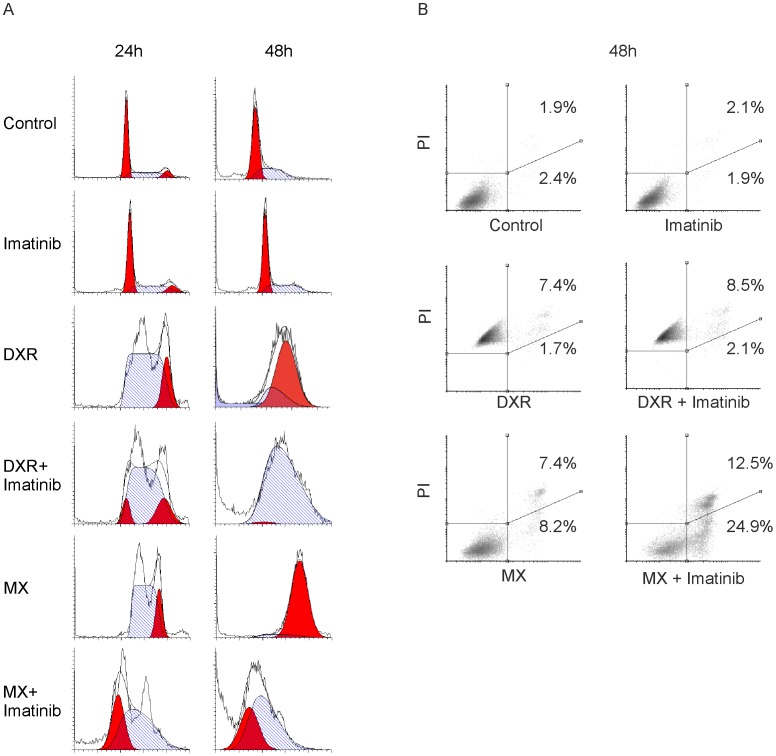
Adding imatinib to MX results in abrogation of G2 phase arrest in HeLa cells and significant increase in apoptosis induction. (A) Cells were treated with MX (0.6 µM), DXR (0.8 µM), imatinib (5 µM) or their combinations for 24 and 48 hours. After treatment, cells were harvested and stained with propidium iodide to quantify DNA content using flow cytometry. Histograms show cell cycle distributions.(B) Annexin-V (X-axis) vs. PI (Y-axis) staining of HeLa cells treated with indicated drugs for 48 h. Percentages indicate the early (lower right quadrant) and late (upper right quadrant) apoptotic fraction. MX and imatinib show a clear synergistic effect in apoptosis induction. The emission spectra of PI and DXR coincide at the FL2 channel, making it impossible to distinguish the early apoptotic population from the late apoptotic population in DXR-treated cells. However, when the right quadrants were added together, there was no difference between DXR and DXR + imatinib populations.

In HeLa cells treated with imatinib alone, at 24 hours, cell cycle distribution was the same as in cells with medium alone, but there were slightly more cells in S phase after 48 hours (Figure 5A). At 24 hours and especially 48 hours, DXR accumulated the cells at G2 phase. Adding imatinib induced S phase arrest in the software analysis; however, at 48 hours with DXR + imatinib, there appeared to be a small G2 population that the analysis software failed to detect. MX-treated cells progressed at 24 hours to S and G2, but the extended incubation after 48 hours showed a clear G2 arrest. Adding imatinib to MX showed at 24 hours a population progressing to G1, indicating abrogation of the arrest. At 48 hours, there were no cells beyond S phase but still also a clear G1 population. This finding suggests that the cells had prematurely entered directly from S phase to mitosis and that the imatinib drove this effect. Alternatively, MX + imatinib may induce a delay from G1/S to G2. However, several unsuccessful mitoses were detected in the time-lapse video of MX+imatinib-treated cells favoring the interpretation that the cell cycle arrest was abrogated (Video S1). We also determined the cyclin B1 levels, a well-acknowledged mitotic marker, in the drug treated cell populations to collect further evidence for the notion that the MX+imatinib co-treated cells are capable of proceeding in cell cycle and entering M-phase. We found that treatment of HeLa cells with DXR, DXR+imatinib, MX and MX+imatinib leads to accumulation of cyclin B1. The protein level of imatinib treated cells was under level of detection similarly to the non-treated controls exhibiting basal level cyclin B1 expression (Figure S5).

Imatinib increased the sub G1 events in MX-treated HeLa cells, partly indicative of apoptosis. The sub G1 population was the largest (38.6%) in the MX + imatinib group at 48 hours. Imatinib also increased DXR-induced sub G1 but to a lesser extent (17.8%) at 48 hours (Table S1). To further analyze the possible apoptosis we stained the cells with annexin V which is a marker of early apoptosis. We found that imatinib significantly increases the proportion of apoptotic cells in MX-treated cells, but failed to detect any increase when imatinib was added to DXR (Figure 5B) Flow cytometry results are in line with the caspase experiment supporting the notion that imatinib with MX is more potent inducer of cell death than with DXR. Imatinib has been previously reported to cause G1 arrest in head and neck cancer cell lines [21] whereas MX and DXR cause G2 arrest [22]. We saw no G1 arrest with imatinib alone.

### Downregulation of c-Abl with siRNA impedes the cytotoxicity of MX + imatinib

**Figure 6:**
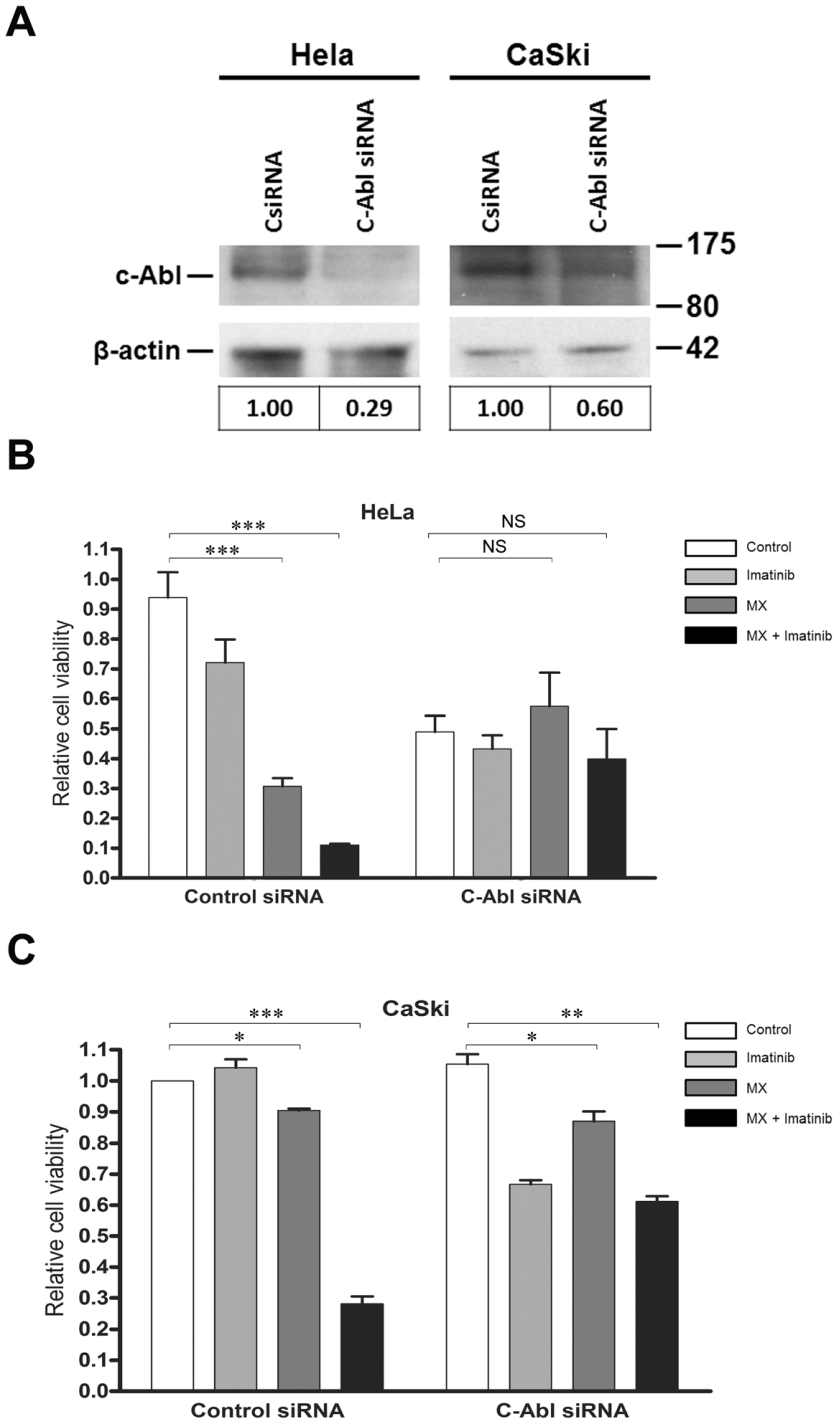
Down-regulation of c-Abl by siRNA counteracts MX induced apoptosis in HeLa and CaSki cells. (A) HeLa and CaSki Cells were transfected with non-targeting or c-Abl siRNA (75 nM). Western blot analysis was performed to examine the effect of siRNA. Level of c-Abl was quantitated and corrected for β-actin level. Values are proportioned to levels of control siRNA for each cell line. (B) HeLa, and (C) CasKi cells were transfected with control or c-Abl siRNA. After treansfection cells were treated with MX (0.6 µM), imatinib (5 µM) and their combination for 48 h. Relative amount of surviving cells was determined by WST-1 assay. Results are from two independent experiments in triplicates. Data are shown as mean ± SD. * p<0.05, ** p<0.01, *** p<0.001. NS, not significant.

**Figure 7:**
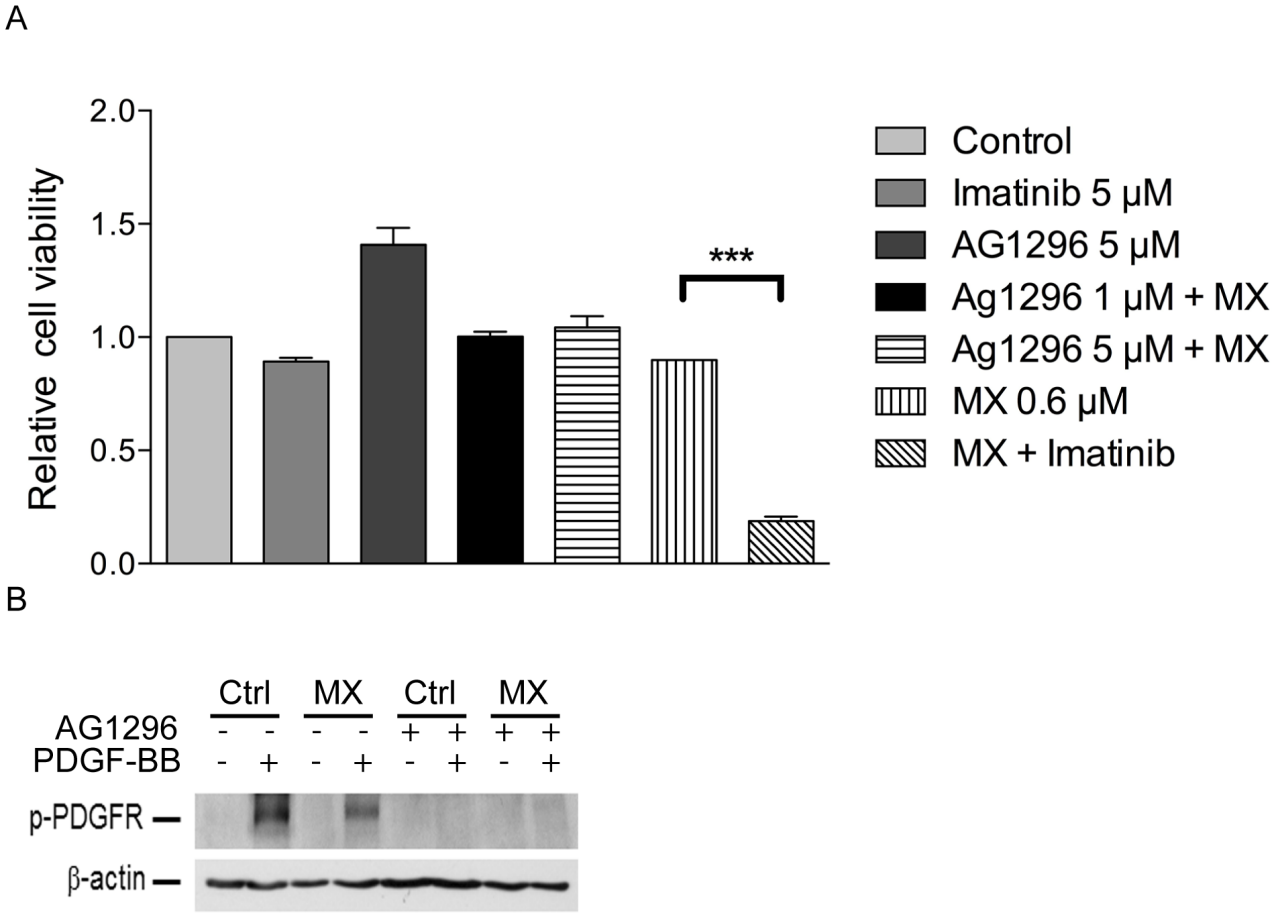
C-kit and PDGF receptor kinase inhibitor AG1296 does not modify the effect of MX. (A) Cells were treated with MX and AG1296 or their combinations for 48 hours and measured with WST-1 assay. Results are from three independent experiments, mean ± SD. *** p<0.001. (B) The effect of AG1296 on PDGFR phosphorylation. HeLa cells were first treated with either medium alone (Ctrl) or 0.6 µM MX for 48 h. Cells were then treated with 5 µM AG1296 for 15 min. Phosphorylation of PGDF receptor was induced by 0.1 µM of PDGF-BB for 10 min.

Imatinib and MX showed an additive effect in reducing the number of control siRNA HeLa cells. When c-Abl was knocked down with siRNA, we found that the proliferation of untreated cells was reduced by 30–50% in repeated experiments (Figure 6, Video S2). Both the growth curves and time-lapse microscopy data show a slight growth inhibition in c-Abl siRNA HeLa cells. However, neither control siRNA nor c-Abl siRNA alone induced cell death. This result suggests that c-Abl is required for the normal proliferation of these cells. Targeting of c-Abl in HeLa cells by siRNA rendered the cells less sensitive to MX. CaSki cells were also less sensitive to MX when c-Abl was downregulated with siRNA, but no inhibition of proliferation was observed. These cells were harder to transfect with siRNA and only 40% efficacy was achieved. This may also explain why the proliferation was not inhibited in these cells. Moreover, the combinatory effect of imatinib was significantly reduced, implicating c-Abl as the pivotal target of imatinib in this outcome. The latter finding is also in line with experiments with other targets of imatinib, PDGF and c-Kit. AG1296 is known to potently inhibit signaling of human PDGF α- and β-receptors as well as of the related stem cell factor receptor c-Kit, at 1 µM and 5 µM concentrations, respectively. AG1296 could not mimic the action of imatinib with MX (Figure 7A). Moreover MX does not have any effect on phosphorylation of PDGF receptor (Figure 7B). Furthermore, the siRNA experiment implied that the kinase-active c-Abl is responsible for cell survival after MX damage. Of importance, this experiment points to different roles for kinase-active and kinase-inactive c-Abl. Kinase-inactive c-Abl appears to be required for apoptosis with MX, but kinase-active c-Abl is a key player for DNA damage repair after MX in HeLa cells.

## Discussion

One of the major findings in this study is that the synergizing effect of imatinib was specifically seen with MX. Previous studies have shown that imatinib may enhance the cytotoxicity of a wide range of chemotherapy drugs in solid tumor–derived cells [13], [23]. Nevertheless, one group found that colon cancer cells become less sensitive to TRAIL-induced apoptosis after imatinib [24]. These reports did not involve testing the possible sensitivity differences between chemotherapeutics in this context. Interestingly, Pinto et al. very recently found in a comparable screening setting that imatinib and MX additively inhibit the proliferation of PC-3 prostate cancer cells [25], [26].

MX is an anthracenedione that targets topoisomerase II. It can also induce DNA intercalation and free radical generation [27]. The effect of imatinib with MX observed in the present study cannot merely be explained by topoisomerase II inhibition because DXR (also a topoisomerase II inhibitor) did not have similar activity with imatinib. The effect of imatinib is linked to its ability to increase DNA damage in target cells, but it did not notably increase the DNA damage induced by DXR in HeLa cells. The difference in these closely related compounds is not entirely clear, but there are some reported differences in these drugs that may be related to the multi-drug resistance (MDR) clearance of the drug.

Several tyrosine kinase inhibitors can selectively modulate MDR-dependent drug efflux [28]. Imatinib has been reported to reverse the resistance to topotecan and SN-38, an active metabolite of irinotecan, and this activity has been attributed to inhibition of the ABCG2 transporter [29]. MX resistance is additionally mediated by transport proteins MRP-1 and ABCB1 [30]. Nilotinib, an imatinib derivative, inhibits the activity of both ABCG2 and ABCB1, leading to enhanced DXR accumulation. Different cells may behave in opposite ways because imatinib has been reported to increase DXR concentration and synergize cytotoxicity in breast cancer cells [31] but not in sarcoma cells [28], [32]. The concentration of the drug in the cell is not likely solely to explain the outcome, because, for example, raising the concentration of the drug does not lead to such a profound p53 accumulation and activity as seen with the MX + imatinib combination (data not shown). Moreover, the cells can be rescued by knocking down c-Abl. Therefore, without c-Abl commitment, the effect is profoundly hampered.

c-Abl knockdown by siRNA rendered HeLa cells resistant to MX. Taken together with the fact that kinase-inactive c-Abl sensitized the cells to MX, this finding indicates that maximal MX cytotoxicity depends on kinase-inactive c-Abl. The results suggest that either 1) kinase-active c-Abl is a direct inhibitor of apoptosis in these cells or 2) kinase-inactive c-Abl is pro-apoptotic in stress conditions. Of relevance, kinase-inactive c-Abl has an important function that is not present in knocked-down cells. It has been shown that in addition to BCR/Abl cell lines, even in solid tumor–derived cells that have increased c-Abl activity, imatinib may inhibit aberrant growth. Moreover, c-Abl activation may either enhance or decrease cell cycle progression in a cell type–specific way or inhibit apoptosis and induce cell death [23], [33]. We saw no difference in cell proliferation or clonogenic growth after imatinib treatment, but c-Abl knockdown diminished proliferation of HeLa cells. This finding suggests that kinase-inactive c-Abl supports cell cycle progression in these cells in unstressed conditions. In fact, a positive mitogenic role for c-Abl has been shown in studies exploiting *abl*-deficient cells [34], [35]. When DNA is damaged, c-Abl is activated, and the cell cycle is stalled. If the catalytic activity is inhibited, the lack of c-Abl enhances cell cycle progression, allowing no time for repair, highlighting the key role of c-Abl in cell cycle control in these cells. These results suggest that kinase activity of c-Abl may either enhance or inhibit proliferation depending on either cell type–specific differences or protein conformation properties and is not simply a binary choice.

c-Abl facilitates a repair checkpoint following moderate DNA damage but promotes death after severe damage. This checkpoint depends on the catalytic activity of c-Abl and is ruptured by imatinib [3]. Checkpoint activation and mitosis block occur especially in cells that have defective replication. Cells lacking the S phase checkpoint with disordered replication eventually die [36]. HeLa cells were arrested in G2 phase after 48 hours of exposure to MX and DXR. Of importance, we found that inhibition of c-Abl kinase activity repressed the G2 phase arrest induced by MX, but not that induced by DXR G1 and the number of annexin positive cells also increased significantly, indicating defective replication that was followed by apoptosis with enhanced caspase 3/7 activation. This finding, together with the comet assay results, suggests a different form of DNA damage recognition that depends on active c-Abl function in the case of MX damage. Consistently, microarray analyses exploring the different outcomes of MX and DXR reveal that there are differences in MX and DXR damage responses (unpublished data). The cell cycle analyses hint at an important connection between c-Abl and cell cycle control for certain chemotherapy drugs. This possibility is in line with c-Abl being a binding partner to a number of substrates linked to cell cycle checkpoint control [4].

After DNA damage, RAD51 is centrally involved in homologous recombination repair and mediates the DNA strand-pairing step. Russell et al. have previously shown that imatinib reduces RAD51 levels in glioma cells, but not in fibroblast cells exposed to X-rays [12]. In the present study, both DXR and MX increased RAD51 levels, and imatinib pretreatment inhibited the increase equally. A similar effect on RAD51 levels suggest that other targets are responsible for the observed c-Abl effect on MX-induced lesions.

p53 is a core node in DNA damage signaling. In HPV-positive cells, its function is knocked down by HPV E6-induced degradation, but it can still be activated after genotoxic insult and play a role in DNA repair and apoptosis [16], [20], [37]. Imatinib alone did not affect p53 reporter activity or alter the protein levels. Imatinib activity also did not increase with cisplatin or DXR. Recently, Chan et al. reported that active c-Abl protects p53 from E6AP-mediated degradation and that imatinib reduces p53 levels [38]. In the present study, we saw no p53 reduction in unstressed conditions, which was supported by the unchanged reporter activity. In contrast, the potentiating effect of imatinib for p53 was profound after MX treatment. These findings indicate a difference between cell lines treated with different chemotherapy drugs in respect to p53 activity after c-Abl kinase inhibition. Second, p53 activation is in line with the observation in comet assays that c-Abl inhibition by imatinib added DNA tailing after MX but not after DXR. Despite the accumulation of transcriptionally active p53, cell survival and clonogenic growth did not alter when p53 was inactivated by overexpression of dominant-negative p53. p73 is a direct substrate of c-Abl and is phosphorylated upon DNA damage [1], [39]. We found no evidence that p73 was responsible for the combined effect in HeLa cells in Western analyses. Upon DNA damage, p73 accumulates in an active c-Abl-dependent manner [40]. We blocked the c-Abl kinase activity by imatinib; therefore, it is not surprising that we saw no p73 dependence for the MX + imatinib effect. Taken together, these results implicate pathways other than p53 or p73 as being involved here in the kinase-inactive c-Abl–mediated apoptosis. The more detailed gene level determinants for the observed cell cycle effects and DNA damage potentiation are of key importance and the work is currently underway.

The role of c-Abl after genotoxic treatment has been very inconsistent. The initial report on mouse embryonic oocytes after cisplatin treatment showed that inhibition of c-Abl activity results in survival [41], although contradicting results have been presented [42]. Wang et al. [9] have reported that Atm-mediated c-Abl activation in response to DSBs further activates both Atm and Atr and their downstream effects, enhancing DNA repair. In contrast, Meltser et al. have reported that inhibition of c-Abl kinase activation after irradiation of mouse embryonic fibroblasts results in higher DSB rejoining and higher survival [43]. It may seem that the form of genotoxic treatment - i.e., chemotherapy or irradiation - may explain the difference in outcomes. However, Fanta et al. have previously shown that inhibition of c-Abl results in genetic instability as a consequence of diminished DNA repair [44]. Our results clearly favor the role of c-Abl as a critical factor in DNA repair and survival in MX-induced damage. Chen et al. have found that ultraviolet irradiation that does not lead to c-Abl activation induces a CUL-4A-dependent ubiquitination and degradation of the DNA-binding proteins DDB1 and DDB2. This process is c-Abl-independent [45]. They then showed that kinase-inactive c-Abl may negatively regulate nucleotide excision repair. This activity would explain the dual role of c-Abl in MX-treated cells, an intriguing possibility that needs validation in subsequent experiments. It is also important to note that the cellular background has a profound influence in this context; therefore, it is relevant that we did not see the effect in primary fibroblasts. c-Abl has a plethora of binding partners and sits at the crossroads of several crucial cellular pathways. Alterations in these genes may have a pivotal impact on c-Abl catalytic activity modulation. The difference in primary cells vs. cancer cells in this respect may also offer unique therapeutic opportunities.

## Conclusion

In this study, we show that c-Abl has a dual role in the damage response after chemotherapy exposure depending on its kinase activity. Furthermore, c-Abl may be a key player in MX-induced genotoxic stress but might be dispensable after several other drug treatments. The kinase-active c-Abl facilitates the repair of the damaged DNA whereas the catalytically inactive c-Abl triggers apoptosis in the afflicted cells. This finding has not been reported before in cells treated with c-Abl-activating damage including chemotherapy or ionizing radiation. The obscure role of c-Abl in BCR/Abl-negative cells has hampered the use of antagonists in solid tumor treatment. It remains to be seen whether different tumor subsets with specific molecular signatures respond to the combined treatment with specific chemotherapy drugs together with c-Abl inhibition.

## Supporting Information

Figure S1 **Imatinib enhances MX induced cytotoxicity also in CaSKi and SiHa cell lines.** Short-term cytotoxicity assay. Results were from three independent experiments, mean ± SD. *** p<0.001.
(TIF)

Figure S2 **Enhancement of MX induced cytotoxicity by imatinib is not p53 dependent.** p53 activity was abolished with either dominant negative p53 (DDp53) or ectopic HPVE6. CMV depicts the empy vector. Treatment duration 48 h. A. Stably transfected HeLa cells B. Stably transfected SiHa cells.
(TIF)

Figure S3 **Imatinib enhances MX induced cytotoxicity in CaSKi and SiHa cell lines with growing in clonal densities.** Targeting of residual p53 activity with ectopic E6 does not rescue SiHa cells from the imatinib enhanced cytotoxicity. Cells were treated in the clonogenic assay with each drug for 12 h. Then, fresh medium was replaced. The concentration of imatinib was 3 µM whereas MX was used in concentrations from 1 nM to 32 nM in experiments done with CaSKi cell line and from 1 nM to 15 nM with SiHa CMV cell line. Results were from three independent experiments, mean ± SD. *** p<0.001.
(TIF)

Figure S4 **p73 protein levels after indicated treatments.** Western blot image from whole cell lysates at 48 h after treatment.
(TIF)

Figure S5 **Cyclin B1 accumulation in HeLa cells treated with imatinib, DXR, DXR+imatinib, MX, and MX+imatinib.** Cyclin B1 protein level was examined with Western blot analysis 48 h after the treatment.
(TIF)

Table S1 **Cell cycle distributions of HeLa cells.** Cell cycle distribution percentages from experiment shown in Fig. 5. Cells were treated with indicated drugs for 24 and 48 h. Cell cycle phase percentages were calculated using ModFit LT cell cycle modelling software. Results are shown as percentages of G1, S and G2/M populations.
(DOCX)

Video S1 **Combination of MX (0.6 µM) and imatinib (5 µM) induces more abnormal mitoses than MX alone in HeLa cells.** Cells were seeded on 96-well plates and images were acquired at 1 hour interval. Bar represents 200 µm.
(MP4)

Video S2 **c-Abl siRNA induces mild growth inhibition but not cell death in HeLa cells.** Cells were seeded on 96-well plates and images were acquired at 1 hour interval. Bar represents 200 µm.
(MP4)
