# Supplementary figures and images for: Inhibition of c-Abl Kinase Activity Renders Cancer Cells Highly Sensitive to Mitoxantrone
Source: PLoS One. 2014 Aug 22;9(8):e105526. doi: 10.1371/journal.pone.0105526 (PMC4141754; doi:10.1371/journal.pone.0105526)

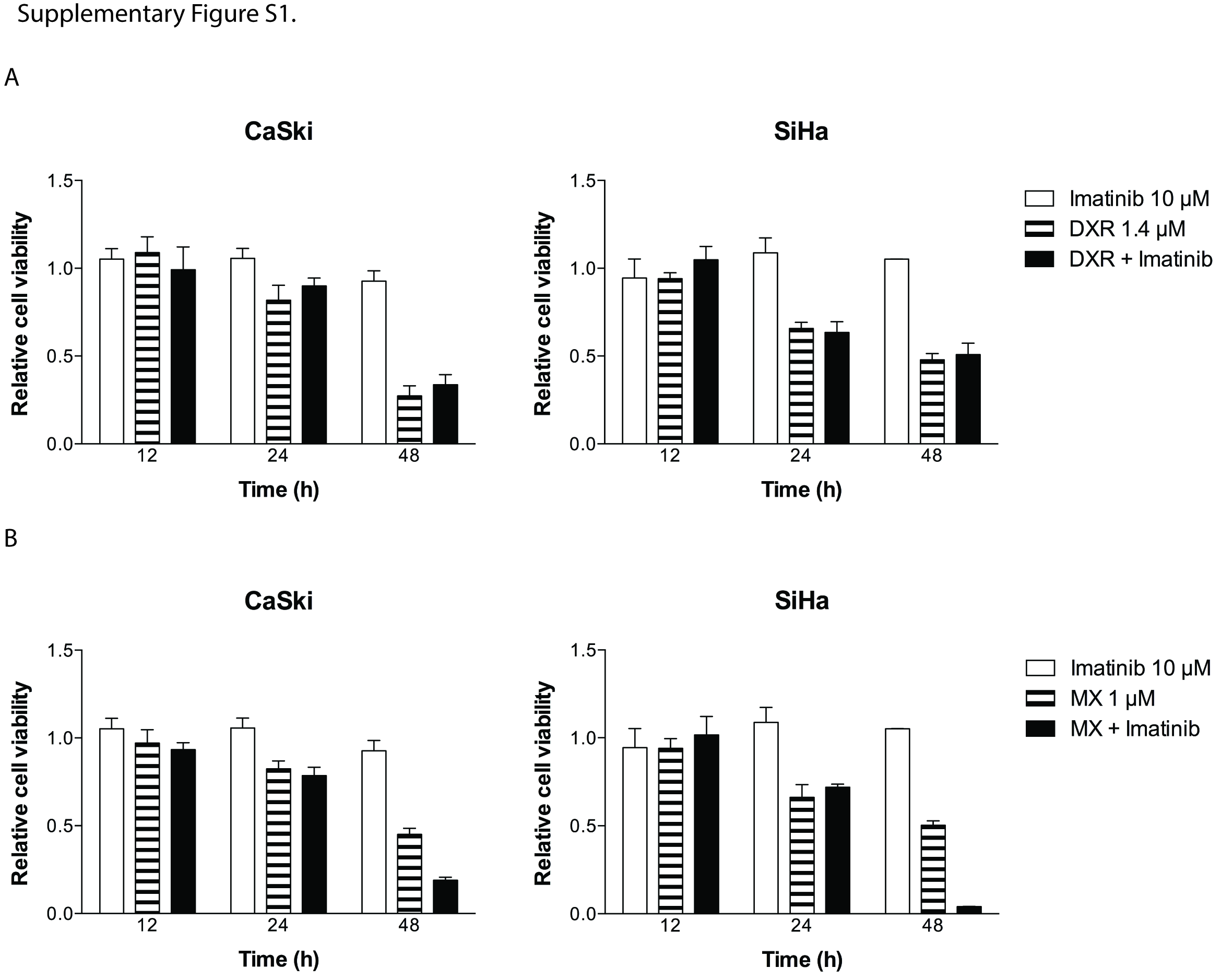

Supplement: Figure S1 — Imatinib enhances MX induced cytotoxicity also in CaSKi and SiHa cell lines. Short-term cytotoxicity assay. Results were from three independent experiments, mean ± SD. *** p<0.001. (TIF) [file pone.0105526.s001.tif]

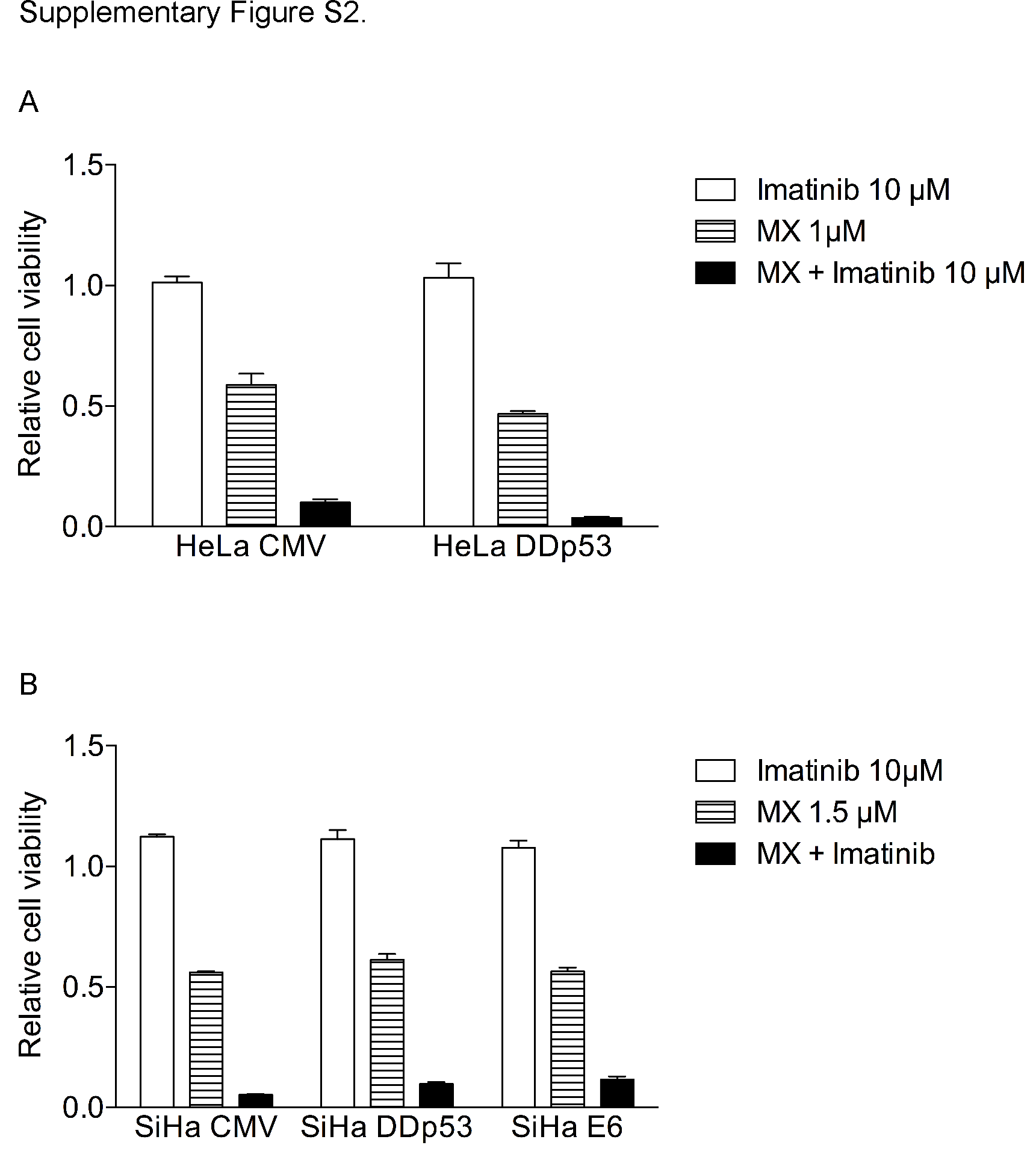

Supplement: Figure S2 — Enhancement of MX induced cytotoxicity by imatinib is not p53 dependent. p53 activity was abolished with either dominant negative p53 (DDp53) or ectopic HPVE6. CMV depicts the empy vector. Treatment duration 48 h. A. Stably transfected HeLa cells B. Stably transfected SiHa cells. (TIF) [file pone.0105526.s002.tif]

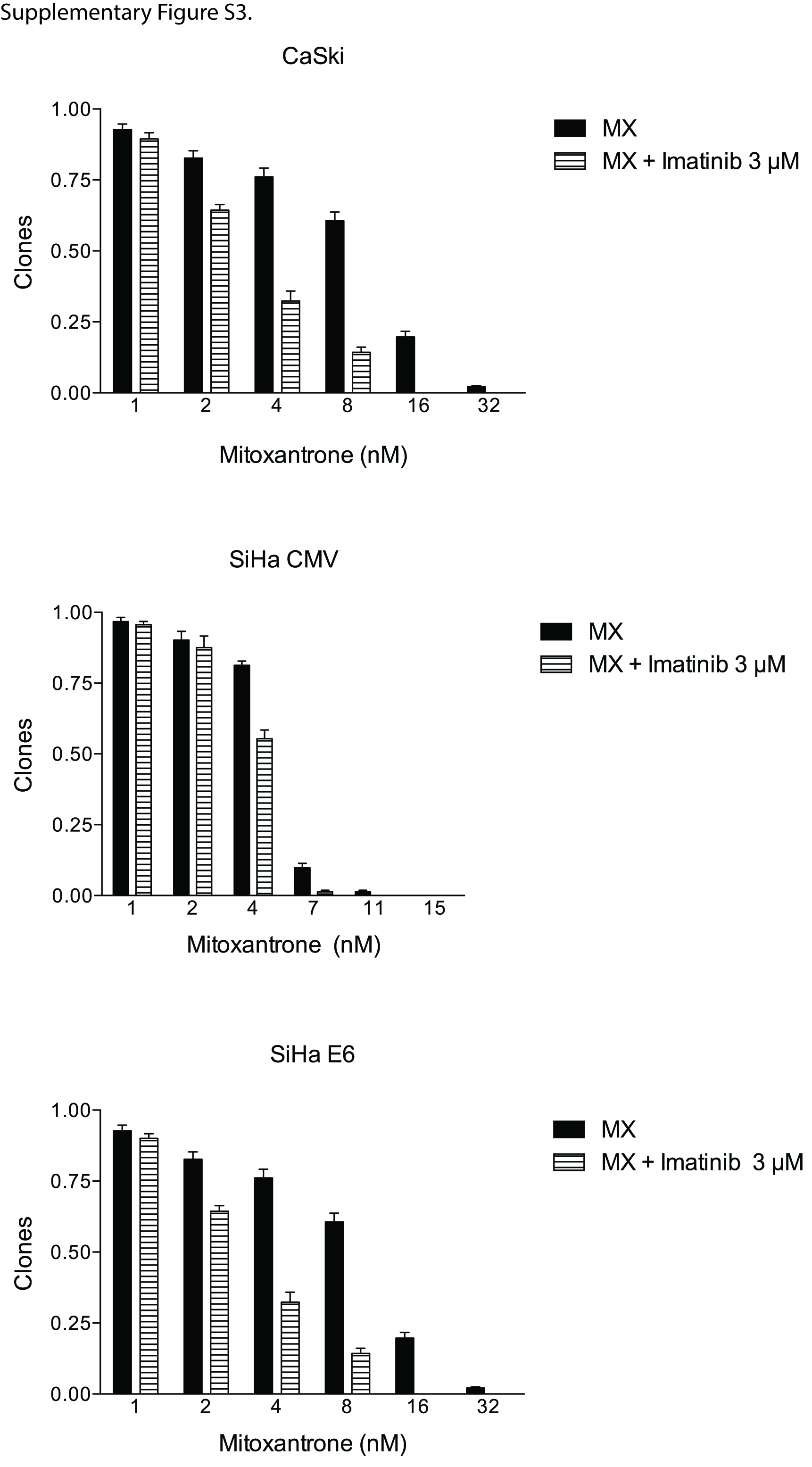

Supplement: Figure S3 — Imatinib enhances MX induced cytotoxicity in CaSKi and SiHa cell lines with growing in clonal densities. Targeting of residual p53 activity with ectopic E6 does not rescue SiHa cells from the imatinib enhanced cytotoxicity. Cells were treated in the clonogenic assay with each drug for 12 h. Then, fresh medium was replaced. The concentration of imatinib was 3 µM whereas MX was used in concentrations from 1 nM to 32 nM in experiments done with CaSKi cell line and from 1 nM to 15 nM with SiHa CMV cell line. Results were from three independent experiments, mean ± SD. *** p<0.001. (TIF) [file pone.0105526.s003.tif]

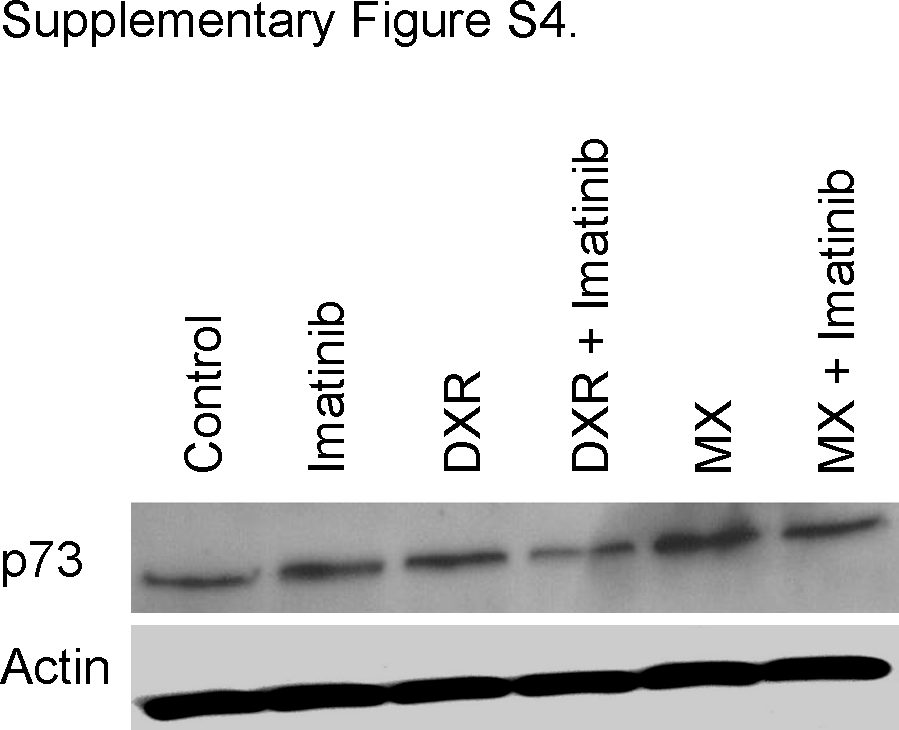

Supplement: Figure S4 — p73 protein levels after indicated treatments. Western blot image from whole cell lysates at 48 h after treatment. (TIF) [file pone.0105526.s004.tif]

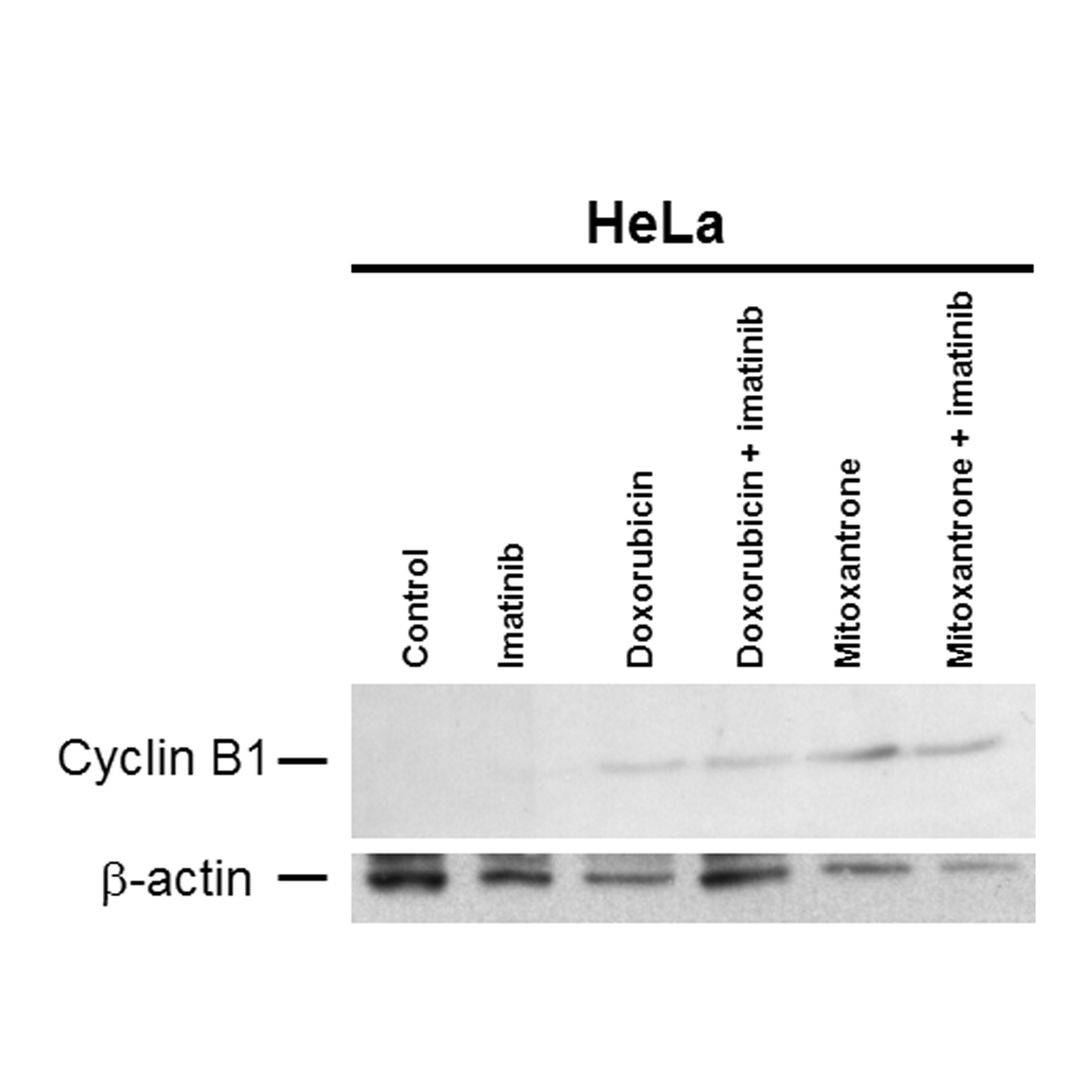

Supplement: Figure S5 — Cyclin B1 accumulation in HeLa cells treated with imatinib, DXR, DXR+imatinib, MX, and MX+imatinib. Cyclin B1 protein level was examined with Western blot analysis 48 h after the treatment. (TIF) [file pone.0105526.s005.tif]
