# Supplementary material for: Inhibition of c-Abl Kinase Activity Renders Cancer Cells Highly Sensitive to Mitoxantrone
Source: PLoS One. 2014 Aug 22;9(8):e105526. doi: 10.1371/journal.pone.0105526 (PMC4141754; doi:10.1371/journal.pone.0105526)
Supplement: Table S1 — Cell cycle distributions of HeLa cells. Cell cycle distribution percentages from experiment shown in Fig. 5. Cells were treated with indicated drugs for 24 and 48 h. Cell cycle phase percentages were calculated using ModFit LT cell cycle modelling software. Results are shown as percentages of G1, S and G2/M populations. (DOCX) [file pone.0105526.s006.docx]

|  | 24h | 48h |
| --- | --- | --- |
|  | G1/S/G2,M (%) | G1/S/G2,M (%) |
| Control | 57/33/10 | 65/35/0 |
| DXR | 0/80/20 | 0/10/90 |
| MX | 0/83/17 | 1/0/99 |
| Imatinib | 53/32/15 | 67/31/2 |
| DXR + imatinib | 21/54/25 | 9/91/0 |
| MX + imatinib | 36/64/0 | 53/47/0 |

**Supplementary Table 1. Cell cycle distributions of HeLa cells**

Cell cycle distribution percentages from experiment shown in Fig. 5. Cells were treated with indicated drugs for 24 and 48 h.  Cell cycle phase percentages were calculated using ModFit LT cell cycle modelling software. Results are shown as percentages of G1, S and G2/M populations.
